# The Educational Gradient in the Adherence to the Healthy Nordic Food Index Among Adult Men and Women in Tromsø: The Tromsø study 2015–2016

**DOI:** 10.29219/fnr.v70.12632

**Published:** 2026-03-04

**Authors:** Nadine El Rashidi, Ainhoa Ugarteche-Perez, Erlend Hoftun Farbu, Raphaële Castagné, Tom Wilsgaard, Sameline Grimsgaard, Marc Chadeau-Hyam, Dragana Vuckovic, Torkjel M. Sandanger, Cyrille Delpierre, Michelle Kelly-Irving, Lola Neufcourt

**Affiliations:** 1 CERPOP-UMR1295, EQUITY Research Team, Inserm, Université Toulouse III Paul Sabatier, Toulouse, France; 2 Department of Community Medicine, Faculty of Health Sciences, UiT the Arctic University of Norway, Tromsø, Norway; 3 Department of Epidemiology and Biostatistics, School of Public Health, Faculty of Medicine, Imperial College London, London, UK; 4 MRC Centre for Environment and Health, School of Public Health, Imperial College London, London, UK

**Keywords:** socioeconomic position, education, social inequalities in health, healthy Nordic diet, healthy regional diet

## Abstract

**Background:**

There is a well-established relation between socioeconomic position (SEP) and diet. People with lower SEPs tend to eat high-calorie, low-nutrient foods, while those with a higher SEP tend to consume foods associated with better health. However, the underlying mechanisms are yet to be understood.

**Objective:**

To examine the association between education and the Healthy Nordic Food Index (HNFI) in men and women in Tromsø, and investigate the role of three intermediate variables: household income, subjective occupational social status, and self-rated health.

**Design:**

Dietary information from Food Frequency Questionnaires were used to construct the HNFI based on six food items and categorised as low, medium, and high adherence. Education and intermediate variables were self-reported. Multinomial logistic regression models stratified by sex were performed to assess the association between education and the HNFI among 8,610 women and 6,896 men aged 40–99 years.

**Results:**

Median intake of all food items increased across categories of the HNFI for all participants. High adherers to the HNFI were slightly older, more educated, had higher household income, perceived their occupational social status as high, and rated their health as good/excellent. We observed an educational gradient in the adherence to the HNFI where men (odds ratios [OR] _TertiaryLong_ 1.92 [95% confidence intervals [CI] 1.47–2.5]) and women (OR _TertiaryLong_ 2.35 [1.94–2.85]) with higher education had higher odds of adhering to the HNFI compared to those with primary education. Household income partly attenuated this gradient in men only.

**Conclusion:**

The association between education and adherence to the HNFI followed an educational gradient, which was partly attenuated by income in men but not in women. Our study highlights potential mechanisms underlying the relationship between education and diet. A deeper understanding of socioeconomic disparities in healthy eating is crucial for enhancing overall nutrition, especially among the socially disadvantaged.

## Popular scientific summary

- This study examined the association between educational level and adherence to a Healthy Nordic Food Index (HNFI).
- Participants with a higher education had higher odds of adhering to the HNFI compared to those with primary education.
- Household income was a potential underlying mechanism in the association between education and adherence to Nordic diet only in men.
- Addressing socioeconomic disparities in healthy eating is crucial for enhancing overall nutrition, especially among the socially disadvantaged.

A well-established relationship exists between socioeconomic factors and health across countries, which is persistent over time (1). A large body of epidemiologic evidence shows that non-communicable diseases such as cardiovascular diseases, cancers, and diabetes mellitus follow a social gradient with health improving incrementally as social position rises (2). However, the mechanisms and processes underlying this association are yet to be understood. Diet is one factor linking social inequalities and disease. Studies consistently show a significant role of socioeconomic position (SEP) in the consumption of different types of food groups and in the intake of micro and macro-nutrients (3). Specifically, individuals with a more disadvantaged SEP are more likely to consume energy-dense, nutrient-poor foods including refined grains, added sugars and fats, and full-fat dairy products (3). Conversely, those with an advantaged SEP are more likely to consume foods associated with better health including whole grains, low-fat dairy products, fish, nuts, fruits and vegetables (3). This association has been demonstrated in several Nordic countries where those with a higher education consumed less fat and more fruits, vegetables and dietary fiber (4, 5). Hence, in order to address socioeconomic inequalities in health, it is important to understand how diet relates to SEP (2, 3).

Diet quality indices have been primarily developed for nutritional epidemiology studies to assess adherence to healthy dietary patterns and their associations with health outcomes (6). Indices measuring the Mediterranean diet, a dietary pattern well-described in the literature, have been evaluated in relation to chronic diseases in Mediterranean and European populations (7, 8). However, there is some controversy regarding adopting this diet in non-Mediterranean countries due to cultural differences in dietary patterns and limited availability of certain foods, raising concerns about environmental sustainability (9). Therefore, identifying other healthy regional diets such as the Nordic diet as an alternative in Nordic countries is important for public health. In Nordic countries, the Nordic diet provides a relevant alternative, emphasising fish, apples, pears, cabbages, root vegetables, berries, and whole grains (10). Studies have shown that the Nordic diet has beneficial effects on health (9) and several *a priori* indices have been developed to measure the adherence to its healthy components (11–13). High adherence to the Healthy Nordic Food Index (HNFI) has been associated with a lower risk of myocardial infarction, reduced mortality, and healthier lifestyles, including greater physical activity and lower alcohol intake (13–15).

Education is a commonly used indicator of SEP which is relatively easy to measure, comparable internationally (16), stable across adulthood (17), and whose collection from participants is often better accepted compared to income or other indicators of wealth (18). It captures the childhood cultural capital of an individual and has been associated with employment opportunities and income, also reflecting material resources (19). The relationship between education and health is complex, and is driven by multiple underlying pathways (16). Two important pathways are the psychosocial and material pathways. A material pathway suggests that individuals with higher education levels may be more likely to secure jobs with healthier working conditions, better health-related benefits and compensation thereby improving affordability of health-promoting living conditions and resources such as fruits and vegetables (20). However, education operates as a probabilistic rather than deterministic indicator of socioeconomic advantage/position, and the strength of this association varies across contexts, and education does not uniformly translate into higher income (16). The psychosocial pathway implies that low education may activate biological mechanisms of endogenous origin through prolonged stress responses related to financial circumstances, poor working conditions (21), or stress related to poor psychological and physiological well-being (22). Psychosocial factors such as subjective occupational social status, defined as the perception of one’s social standing in the social hierarchy, has been associated with different health outcomes (23).The experience of being lower in the social hierarchy is a stressful one, and in turn may predispose individuals to poorer health trajectories (24). Another factor that might reflect psychosocial resources is self-rated health. It is a commonly used measure of perceived health that provides an overview of one’s health status by asking a single-item question and is associated with various health outcomes and behaviours (25, 26). Physiological stress related to living and financial circumstances may lead to anxiety and depression. Over time, worsening physical and mental health resulting from these stressful conditions may become additional stressors and be associated with poorer diet quality (26).

Although one study reported educational differences in adhering to the HNFI descriptively in a sample of women (27), the relationship between education and adherence to a Nordic diet has not been investigated in the general population in Norway. In order to better understand the needs of the population to adopt a healthier diet and design successful public health policies that mitigate inequalities in health, it is imperative to consider the potential mechanisms at play in any relationship between education and adherence to a Nordic diet (28). The aim of this study is to: 1) examine the association between education and the HNFI separately in men and women who participated in the seventh wave of the Tromsø Study, and 2) investigate the contribution of three potential intermediate variables to this association-namely household income (material pathway hypothesis), subjective occupational social status and self-rated health (psychosocial pathway hypothesis) using attenuation analyses.

## Materials and methods

### Participants

**Fig. 1:**
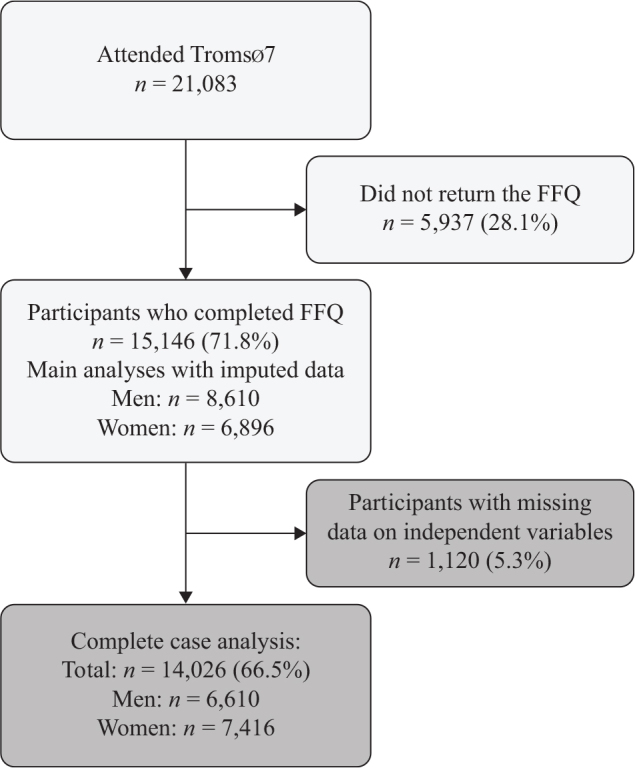
Flow chart of the study population: the Tromsø study 2015–2016. FFQ, food frequency questionnaire.

The Tromsø Study is a population-based cohort study based in the municipality of Tromsø, in northern Norway (29). To date, seven surveys have been conducted with 7–8 years apart. In this study, we used the cross-sectional data from the seventh survey conducted in 2015–2016. Data collection included questionnaires, physical and clinical examinations, and blood and urine sampling (30). Inclusion criteria for Tromsø7 were to be residents of the municipality of Tromsø and to be 40 years and older. In our study, we excluded participants who did not complete the food frequency questionnaire (FFQ). Thus, among 32,591 eligible people invited by mail, a total of 21,083 women and men aged 40–99 years attended Tromsø7 (response rate 65%) (Fig. 1), among whom 15,146 participants returned their FFQ (72% of Tromsø7 participants and 46% of those originally invited).

The study was conducted in accordance with the Declaration of Helsinki Ethical Principles involving human subjects. The ethical approval of the seventh survey was done by the Regional Committee of Medical and Health Research Ethics North and the Norwegian Data Protection Authority. Participants were asked to sign consent forms and data were securely stored in an information technology (IT) system approved by the Norwegian Data Protection Authority (29).

### Variables

#### Dependent variable: the HNFI

A previously validated FFQ (31) was used to collect information about diet during the past year. The FFQ consisted of questions about 261 different food items, dietary supplements, drinks, and meals. Intake of a variety of foods was mapped by using questions on frequency and amount of intake. The intake of food was calculated based on the Norwegian food composition using the calculation system Kostberegningssystemet (KBS) at the University of Oslo (32).

Our outcome measure is the HNFI (13) adapted to the dietary information in the Tromsø study. The original HNFI was first developed in 2011 by Olsen et al. in Denmark (13) based on traditional Nordic food and included six food items: fish, cabbage, root vegetables, apples and pears, oatmeal, and rye bread. In our study, the HNFI was applied as closely as possible to the original index and the calculation of each of the six food items depended on the availability of relevant questions in the FFQ. The total number of grams of each of the six food items were obtained by the addition of these questions. The HNFI included in grams/day: 1) the intake of fish (based on 11 questions related to fish and fish spreads from the FFQ), 2) cabbage (4 questions on the intake of cabbage, cauliflower, broccoli, and brussels sprouts), 3) root vegetables (2 questions about carrots and swede), 4) apples and pears (2 questions), 5) oatmeal (3 questions about oats and cereal oats), and finally 6) whole-grain bread. The food items in our study are the same as those used by Olsen except for the rye-bread category, which was replaced with a whole-grain bread category, as it was not initially asked in the FFQ and because of cultural differences in food between Norwegian and Danish diets (27) (3 questions on whole-grain bread 50%, whole-grain bread 50–100%, and whole-grain crispbread) (Supplementary Table 1).

For each of the six food items, and similar to Olsen et al., one point was given for an intake above the sex-specific median for the sample. For men, the median intake of oatmeal was 0 because 50% of the participants did not consume oatmeal, so, one point was given to male participants with any intake of oatmeal (27). The total score ranged between 0 and 6 and was then categorised into three groups whereby a score between 0 and 1 indicated a low adherence to the HNFI, a score between 2 and 3 indicated medium adherence, and a score between 4 and 6 indicated high adherence (13).

#### Exposure: education

The highest educational level achieved by the participants was self-reported (4 categories: primary/partly secondary education [up to 10 years of schooling]; upper secondary education [at least 3 years]; tertiary education, short [college/university less than 4 years] and tertiary education, long [college/university 4 years or more]).

#### Intermediate variables

*Household income.* Annual household total taxable income (including social benefits) was self-reported by the participants in eight income brackets: 1) Less than 150k Norwegian Krone (NOK), 2) 150k–250k NOK, 3) 251k–350k NOK, 4) 351k–450k NOK, 5) 451k–550k NOK, 6) 551k–750k NOK, 7) 751k–1,000k NOK and 8) more than 1,000k NOK). For this analysis, these categories were then collapsed into four groups based on quartiles: low (combining categories 1–4): ≤ 450k NOK, lower-middle (combining categories 5 and 6): 451k–750k NOK, upper-middle (category 7): 751k–999k NOK and high (category 8): more than 1,000k NOK.

*Self-rated health.* Participants’ self-rated health was assessed using answers to the question: ‘How do you in general consider your own health to be?’ (5 categories: very bad, bad, neither good nor bad, good, and excellent). Because of the low number of participants in the ‘very bad’ category, categories ‘very bad’ and ‘bad’ were collapsed, leading to a four-category variable.

*Subjective occupational social status.* Participants were asked to pick the social status that best describes their occupation (actual or latest if not employed at the time of the questionnaire) among the following options: very high social status, fairly high, neither high nor low, fairly low, and very low. Due to the low number of participants in the ‘very low’ group, we collapsed the categories into three: low (combining fairly low and very low), neither high nor low, and high (combining fairly high and very high).

#### Confounders: age

Age was used as a continuous variable and was adjusted for in the multinomial logistic regression models examining the association between education and adherence to the HNFI.

### Data analysis

Because of sex differences in the medians for each of the food items in the HNFI (33), all analyses were stratified by sex. For the descriptive analysis, we present means (standard deviation [SD]) or n (%). In bivariate analyses, we used the Kruskal–Wallis test and the chi-squared test to test differences across adherence categories.

To reduce potential selection bias in carrying out complete case analyses, we performed multiple imputation of the missing independent variables (*n* = 1,120 participants with imputed data) (Fig. 1): education, household income, subjective occupational social status, and self-rated health using the multivariate imputation by chained equations (MICE) procedure. Multiple imputations were run based on the assumption that data were missing at random. After five iterations, convergence was achieved resulting in 20 imputed datasets. The main analyses were conducted on the imputed dataset. Complete case analyses were conducted as sensitivity analyses and are presented in Supplementary Tables 2a and 2b.

**Fig. 2:**
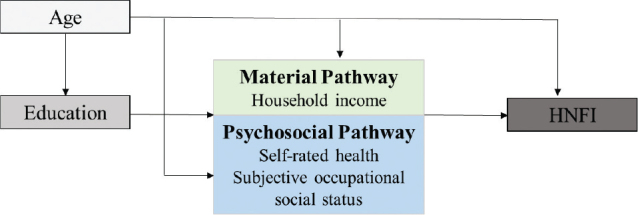
Conceptual framework of the association between education and the Healthy Nordic Food Index and the underlying material and psychosocial pathways. HNFI, Healthy Nordic Food Index.

A multinomial logistic regression was used to analyse the relationship between education and adherence to the HNFI comparing medium and high adherence to the low adherence reference category. Based on our conceptual framework (Fig. 2), five models were considered: model 1 was adjusted for age only; models 2, 3, and 4 were further adjusted for household income, subjective occupational social status, and self-rated health, respectively. Model 5 was adjusted for age and all three intermediate variables together (Fig. 2). The adjustment by blocks of intermediate variables allowed us to assess their contribution to the association. The estimates were presented as odds ratios (OR) with 95% confidence intervals (95% CI). All analyses were performed using R Studio version 2022.07.2+576.

## Results

### Characteristics of the study population

As highlighted in the flow chart (Fig. 1), participants were excluded if they did not return their FFQ (n = 5,937). In the paper, we present the results of the analyses conducted on the imputed data which included a total of 15,146 participants. Results of the complete case analyses by sex are presented in Supplementary Tables 2a and 2b in men and women, respectively.

Among women, there were more participants in the medium or high adherence category compared to the low adherence category, while among men there were more participants in the high adherence category compared to the low and medium adherence categories (Table 1). Half of the total participants received a short or long tertiary level education. A higher proportion of men than women reported high income (26.3% *vs.* 19.9%) and high subjective social status of their occupation (47.0% *vs.* 36.6%). A higher proportion of women than men reported excellent self-rated health (16.5% *vs.* 13.0%).

**Table 1:** Characteristics of study sample by sex: the Tromsø study 2015–2016

Variables	Total (*n* = 15,146)	Men (*n* = 6,986 – 46%)	Women (*n* = 8,160 – 54%)	*P*-value*
**HNFI, *n* (column %)**				
Low	2,530 (16.7)	572 (8.1)	1,316 (16.1)	
Medium	6,453 (42.6)	2,818 (40.3)	3,481 (42.6)	
High	6,163 (40.6)	3,596 (51.4)	3,363 (41.2)	
**Age (years), mean (SD)**	58.7 (11.3)	59.1 (11.3)	58.4 (11.3)	<0.001
**Education, *n* (column %)**				<0.001
Primary/Partly secondary	3,534 (23.3)	1,536 (22.0)	1,998 (24.5)	
Upper secondary	4,061 (26.8)	2,035 (29.1)	2,026 (24.8)	
Tertiary/Short	2,881 (19.0)	1,499 (21.5)	1,382 (16.9)	
Tertiary/Long	4,415 (29.1)	1,804 (25.8)	2,611 (32.0)	
Missing	255 (1.7)	112 (1.6)	143 (1.8)	
**Household income, *n* (column %)**				<0.001
Low	3,395 (22.4)	1,279 (18.3)	2,116 (25.9)	
Lower-middle	4,385 (29.0)	2,035 (29.1)	2,350 (28.8)	
Upper-middle	3,290 (21.7)	1,678 (24.0)	1,612 (19.8)	
High	3,460 (22.8)	1,839 (26.3)	1,621 (19.9)	
Missing	616 (4.1)	155 (2.2)	461 (5.6)	
**Subjective occupational social status, *n* (column %)**				<0.001
Low	975 (6.4)	385 (5.5)	590 (7.2)	
Neither high nor low	7,522 (49.7)	3,174 (45.4)	4,348 (53.3)	
High	6,269 (41.4)	3,282 (47.0)	2,987 (36.6)	
Missing	380 (2.5)	145 (2.1)	235 (2.9)	
**Self-rated health, *n* (column %)**				<0.001
Bad	759 (5.0)	309 (4.4)	450 (5.5)	
Neither good nor bad	3,867 (25.5)	1,784 (25.5)	2,083 (25.5)	
Good	8,145 (53.8)	3,947 (56.5)	4,198 (51.4)	
Excellent	2,254 (14.9)	907 (13.0)	1,347 (16.5)	
Missing	121 (0.8)	39 (0.6)	82 (1.0)	

SD, standard deviation; HNFI, Healthy Nordic Food Index.

* *P* values for categorical variables were obtained with the chi-squared test, and for numerical variables with the Kruskal–Wallis test. A *P*-value was not computed for differences between men and women in HNFI due to sex-specific cut-offs in the score calculation.

The intake of the HNFI food items among all three adherence categories is presented in Table 2 whereby the median intake significantly increased for all items across the adherence categories for both men and women. The biggest difference between the three adherence categories was observed in the whole-grain bread for men and in the apples and pears for women. Women consumed more fruits and vegetables (root vegetables, cabbage, and apples and pears) in total and in each adherence categories of the index when compared to men who consumed more bread and fish.

**Table 2:** Median consumption of food items (grams/day) in the Healthy Nordic Food Index by adherence category and stratified by sex: the Tromsø study 2015–2016

HNFI adherence categories	Median of food items in the HNFI in grams/day (IQR)					
	Fish	Root vegetable	Cabbage	Whole-grain bread	Apples & pears	Oatmeal
**Men**						
Total (*n* = 6,986)	66 (58)	47 (50)	19 (35)	160 (92)	32 (64)	0 (15)
Low (*n* = 572)	31 (29)	10 (18)	5 (10)	98 (54)	10 (16)	0 (4)
Medium (*n* = 2,818)	51 (39)	25 (36.7)	11 (18)	140 (84)	21 (44)	0 (11)
High (*n* = 3,596)	87 (52)	60 (45)	34 (41)	180 (87)	65 (78)	2 (21)
*P* value*	<0.001	<0.001	<0.001	<0.001	<0.001	<0.001
**Women**						
Total (*n* = 8,160)	51 (45)	50 (64)	27 (47)	118 (76)	54 (81)	6 (28)
Low (*n* = 1,316)	29 (24)	21 (20.2)	10 (14)	89 (52)	16 (25)	0 (3)
Medium (*n* = 3,481)	44 (37)	45 (41)	21 (34)	112 (74)	36 (60)	3 (20)
High (*n* = 3,363)	70 (42)	72 (52)	48 (52)	132 (70)	75 (63)	16 (38)
*P* value*	<0.001	<0.001	<0.001	<0.001	<0.001	<0.001

IQR, Interquartile range; HNFI, Healthy Nordic Food Index.

* P < 0.001 for Kruskal-Wallis test across the low, medium and high adherence categories for both sexes respectively.

The distribution of the confounding and intermediate variables according to the HNFI score categories for men and women is presented in Supplementary Table 3. Both men and women with high scores of the HNFI were older, perceived their occupational social status as high, and rated their health as good or excellent. Men with a high score of the HNFI were more likely to have a higher education. However, educational level did not differ between medium and high adherers in women.

### Education and the HNFI

**Fig. 3:**
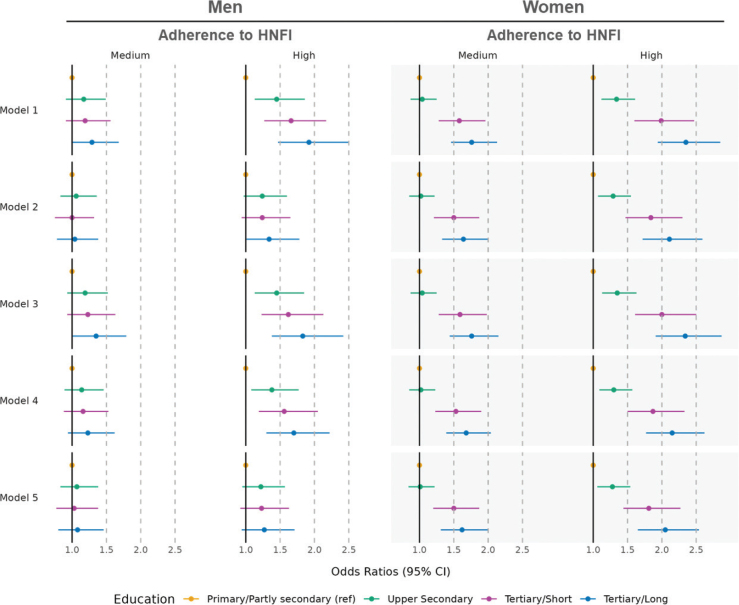
Multinomial logistic regression models of the association between education and adherence to the Healthy Nordic Food Index by sex, men: *N* = 6,610 & women, *N* = 7,416: The Tromsø Study 2015–2016. HNFI, Healthy Nordic Food Index.

Figure 3 presents the results of the multinomial logistic regression models where belonging to the medium or high adherence categories is compared to belonging to the low adherence category. In men, there is a weak indication of an educational gradient in the odds of being in the medium adherence category of the HNFI (model 1). However, a strong educational gradient was visible in the odds of being in the high adherence category (model 1), where men with a tertiary education, had greater odds of being in the high adherence category compared to men with primary education (OR 1.92 [95% CI 1.47–2.50]). This pattern was also observed in women, with higher odds of being in the medium and high adherence categories compared to the low adherence category, especially for tertiary short and long education.

To investigate the role of the two main potential pathways in the relationship between education and the HNFI represented by three intermediate variables, four additional explanatory multinomial logistic regression models were run for both, men and women. Supplementary Tables 4a and 4b present the ORs and corresponding 95% CI for men and women.

For men, the association between education and the odds of being in the medium and high HNFI adherence categories decreased for all educational levels when adjusting for household income (model 2). For instance, the OR of being in the medium adherence category for those with an upper secondary level decreased from 1.17 [95% CI 0.91–1.49] to 1.06 [0.83–1.36] and from 1.45 [1.13–1.86] to 1.24 [0.97–1.6] for the high adherence category. When further accounting for household size to better reflect disposable income per household member in Supplementary Table 5, we observed the same trends as in models 2 and 5 in Supplementary Table 4. This was also observed in model 4 when self-rated health was added, where a slight decrease in the association between education and high adherence to the HNFI was observed. This decrease in effect size however, was not observed in model 3 for the medium adherence category when subjective occupational social status was adjusted for, and slightly observed in the high adherence category for tertiary short and long education. The OR of being in the medium or high adherence categories of the HNFI decreased across all educational levels once all intermediate variables had been added to the model (model 5).

For women, a small decrease in the effect size of the association between education and the odds of being in the medium and high adherence categories of the HNFI was observed when household income (model 2) and self-rated health (model 4) were added. This decrease was more pronounced in the odds of being in the high adherence category. However, when adjusting for subjective occupational social status (model 3), the association between education and adherence to the HNFI remained largely unchanged. When adjusting for all intermediate variables simultaneously (model 5), the effect size of the association between education and both the medium and high adherence to the HNFI categories decreased across all educational levels.

Supplementary Table 2 shows the associations between education and the outcome in complete case analyses by sex. The results of these analyses show the same associations. However, the educational gradient seemed to be less pronounced especially within high adherence category of the HNFI for men in all models.

## Discussion

This study aimed to examine the relationship between education and adherence to the HNFI in a sample of participants to the seventh wave of the Tromsø Study and to exploratively investigate potential material and psychosocial mechanisms by adjusting for intermediate variables. To our knowledge, this is the first study to examine SEP in relation to the HNFI in this specific context, using detailed and culturally relevant dietary data. We found that adherence to a healthy Nordic diet was socially patterned across educational categories among men and women, within a population-based sample of people from Tromsø, Northern Norway. We also found that the relationship between education and adherence to the HNFI was modified by intermediate variables and differently for men and women. In men, the association was affected by household income while in women, the relationship was not affected by any of the intermediate variables. These findings suggest that potential material and psychosocial mechanisms may be involved in the relation between education and diet, and that these may vary according to sex.

### Educational gradient in the adherence to the HNFI

The results show that there is an educational gradient in the odds of adhering to the HNFI and this gradient is more pronounced for those belonging to the high adherence category of the HNFI in men and in both medium and high adherence categories in women, where participants with a long tertiary education had higher odds of adhering to the HNFI than those with a lower education. Our results are based on the analysis of a single wave of the Tromsø Study and are in line with previous studies that used education and other indicators of SEP such as income and occupation in relation to multiple measures of high quality diets in different groups by age and sex (3). Research has repeatedly shown that higher quality diets are consumed by more educated and affluent people (3). Consistent with descriptive findings from the Norwegian Women and Cancer Study (NOWAC) cohort, we found that adherence to the HNFI was socially distributed and especially when looking at the high adherence categories in both men and women (27). These findings point to a greater likelihood of a high level of adherence among more educated participants. Our findings add to a substantial body of existing research demonstrating social inequalities in diet by investigating the educational gradient in a healthy Nordic diet. Hence, it is key to further understand, from a public health perspective, the potential underlying mechanisms in the relationship between SEP and diet. Numerous individual and environmental level factors have previously been identified as partial mediators of the relationship between SEP and diet including: access to healthy food retail, nutrition knowledge, stressors and psychological resources, and food cost or affordability (3, 34, 35).

### Intake of HNFI food items

The median intake of the HNFI food items in this sample appears higher compared to two other Nordic cohorts: The Diet, Cancer and Health cohort in Denmark and the NOWAC cohort in Norway (27, 36). This could be due to differences in intake between the respective sample populations or different assessments and number of food items included in calculating the score. The food item distributions across HNFI adherence categories in women in our sample were similar to findings in the NOWAC study, where those with high adherence to the HNFI had a significantly higher intake of all six food items compared to medium and low adherers (27).

Differences in the intake of the HNFI food items were observed between men and women. Consistent with prior research examining gender differences in fruit and vegetable consumption, women in this study consumed more fruits and vegetables (37). Consumption of fish did not differ between men and women in other studies contrary to our study (38, 39), possibly indicating that our findings may be specific to our population living in Northern Norway. For example, fish has been associated with male identity in fishing communities (40).

### Potential underlying mechanisms

To further explore the underlying mechanisms of the relationship between education and adherence to the HNFI, we adjusted for material and psychosocial intermediate variables. Observed changes in the effect size of the relationship between education and HNFI adherence could indicate a mechanism that might warrant further examination through formal mediation analyses.

While some studies have shown how psychosocial factors such as financial stressors and psychological distress could be the underlying factors as to how education influences eating behaviour, we found no clear evidence of a potential mediating role of self-rated health and subjective occupational social status on adhering to the HNFI. Our findings showed very slight attenuation in the association between education and adherence to the Nordic diet after adjusting for these factors contrary to a study conducted by Mulder et al. This study of 3,050 Dutch participants with a mean age of 44 years found that financial stressors and perceived health status were mediating stressors between education and healthy behaviours (35). This observation may be due to differences in countries or age between the two populations (our study sample has older participants) and may be driven by material resources especially during later phases of adulthood when opting for a healthy diet (41).

In women, the association between education and adherence to the HNFI was slightly attenuated after adjustment for all intermediate variables, suggesting that the role of education in the social patterning of adherence to a healthy Nordic diet in this population in Tromsø was not explained by any of the tested intermediate variables. This strong influence of education on diet may be mediated by other factors not measured in this study. A qualitative study of 56 women from Australia investigating why women from disadvantaged socioeconomic groups have poorer dietary behaviours showed that women from advantaged socioeconomic groups more often referred to concerns for their health when making food choices than women from disadvantaged socioeconomic groups (42). Barker et al. (43) described in a sample of 240 women in Southampton, United Kingdom (UK), how women with lower education levels have poorer quality diets than women with higher education levels because they are less involved in food and meal preparation. Our findings alongside these works may point to a behavioural pathway (16) operating through performative gender roles linked to women’s health-seeking behaviours. Firstly, it is well-established that women are more knowledgeable about food and nutrition, are better health information seekers, and are more compliant to healthy dietary behaviours compared to men (44, 45). Secondly, women are more familiar with dietary guidelines than men which is likely to be a consequence of their greater exposure to food and food-related processes throughout their life course and different media influences (40). From early on, females are socialised into care-giving roles which bring them into constant contact with food. Later on in life, women are the principal providers and servers of food and are considered the principal guardians of their family’s health and the provision of nutritious food is considered to be important to them (43, 44).

In men, among all mediators, household income attenuated the association between education and adherence to the HNFI the most, suggesting that education influences adherence through a material pathway. One possible explanation to these findings may be related to the cost of food: foods of higher nutritional quality have been demonstrated to cost more per calorie (46). There is consistent evidence that food cost (47) could explain dietary inequalities and especially in the context of Norway where the cost of food such as fruits and vegetables are higher compared to other European countries (48). Hence, higher income groups can better support the affordability of healthier foods and possess greater financial freedom to take health aspects into account. This interpretation is consistent with conclusions reached by Turell et al. in a study of 1,003 Australian households examining how the effect of education on food purchasing was substantially attenuated after adjusting for household income (49). However, we did not observe the same influence of household income on the relationship between education and adherence to the Nordic diet in our study in women. Since the relationship between education and income was similar in both men and women, this may suggest that income extends beyond diet-specific factors such as the affordability of healthy food and may possibly be reflecting other social dynamics including social distinction in men. The mechanism of social distinction has been tested in the relationship between SEP and the consumption of food products particularly rich in anti-oxidants and vitamins in a population-based Dutch study of 2,812 participants. Those in a higher SEP adopt dietary patterns by which they can set themselves apart from lower SEP groups (50). In a welfare state such as Norway, where people can afford various consumer goods, health-related lifestyle might be a way for people to show their social status.

The potential mechanisms at play in the relation between education and adherence to the HNFI differed between men and women, and could suggest that socially constructed gender roles contribute to differences in adhering to a healthy diet between the sexes.

### Strengths and limitations

The main strengths of this population-based study included a large sample size, a previously validated FFQ for the development of the food index (31), and the inclusion of food items associated with health outcomes for constructing the index, adding to the validity of the tool. Missing data for education, household income, subjective occupational social status and self-rated health were imputed, which may reduce the possibly biased estimates from the complete case analyses. Furthermore, the study included participants from urban and rural areas of Tromsø.

The study also has limitations. First, we restricted our study to one wave of the Tromsø Study; therefore, our analyses were on cross-sectional data which prevents drawing conclusions about causality. Although our study was cross-sectional and the relationship between education and the HNFI was not followed in a longitudinal design, we used educational attainment which typically becomes fixed in the late teens to early 20s and remains relatively stable overtime. It therefore likely reflects an early/young adulthood SEP that temporally precedes the intermediate and outcome variables, which provides some directionality when interpreting the findings. This indicator is widely used in research on dietary inequalities and minimises concerns about reverse causation.

However, this directionality is less clear between the three intermediate variables included in our study and the HNFI, therefore related findings should be interpreted with caution. In particular, the association between self-rated health and the HNFI may be bidirectional as a healthy dietary pattern may improve self-rated health while poorer self-rated health may lead to dietary changes either improvement or deterioration in diet quality.

Second, our sample might be subject to selection bias. Only participants who responded to the FFQ were included in the analysis. Differences were observed in age and sex, where participants who returned the FFQ were older and more likely to be females than those who did not return the FFQ – who were excluded from analysis – but did not differ in educational level. We also acknowledge that, as with all observational studies, bias introduced through voluntary participation may be an issue, and the sample of participants to the wave 7 of the Tromsø Study may differ from the general population of people residing in the catchment area of Tromsø.

While our study provides valuable insights into the intake of HNFI and its association with socioeconomic factors, it is important to consider the context of the general Norwegian population. Our sample exhibits a higher proportion of individuals with higher educational attainment compared to national statistics, which may influence the observed disparities in dietary habits. Given that our sample is more educated than the general population, these disparities are likely to be more pronounced in the broader Norwegian context. Hence, our sample is not representative of the general population in Northern Norway, which should be considered when interpreting the intake of the HNFI food items. Also, the results from the complete case analysis differed slightly from those of the imputed analysis, which may also suggest a potential selection bias. Third, FFQs are prone to recall bias because participants are usually asked to report their intake retrospectively. In addition, social desirability bias would imply that some participants might over-report their intake of healthy food items, such as vegetables and fruits. Also, some food items in the HNFI (fish, oatmeal and whole-grain bread) have not been individually validated in published analyses. Fourth, in the seventh survey of the Tromsø Study, participants’ education was self-reported which is a measure that can lead to exposure misclassification and consequently bias the study’s results. However, a validity study compared self-reported education in the Tromsø Study among three age groups (40–52, 53–62, 63–99 years) with educational level records of Statistics Norway and found self-reported education was adequately complete and correct for research purposes, with fair values of weighted kappa in all age groups and in both, men and women (51). Fifth, we used a food-based index that measures the adherence to an intake of certain food items from the Nordic region that have shown to have health benefits. However, we understand the plausible limitations of the ability of the index to reflect the totality of the participants’ diet. In other words, high adherers to the HNFI might be simultaneously consuming foods high in salt and trans-fatty acids. Hence, development of additional indicators specific to the Nordic context to adequately reflect the intake of salt, sugar and saturated fats is required. Finally, our study has highlighted that different potential mechanisms may underly the relationship between education and adherence to a healthy diet, namely between men and women. Our approach was exploratory rather than confirmatory. To examine these mechanisms in further depth, it would require carrying out formal mediation analyses which was beyond the scope of this research work. Nevertheless, we consider this work as an important preliminary step, which lays the groundwork for future studies employing more advanced methods such as structural equation modelling to formally quantify direct and indirect effects.

## Conclusion

This study shows that people with a higher level of education were more likely to adhere to a traditional Nordic diet, measured by the HNFI. Including household income in the model weakened the relationship between education and adherence to the Nordic diet in men only. Our study shows that different potential factors and mechanisms could be involved in the pathway linking education and adherence to diet, especially in relation to gender. In order to improve diet quality and achieve good nutrition for all, and particularly among those who are more disadvantaged, a more comprehensive understanding of the drivers of socioeconomic inequalities in adherence to a healthy diet is required. Our study established a relationship between education and adherence to a healthy Nordic diet offering insights that can inform future public health strategies aimed at reducing dietary inequalities in Nordic populations. However, to understand the origin of this observed relationship, future work taking a life course perspective to examine the family sociocultural background and childhood circumstances that may influence both education and dietary habits would provide insight.

## Supplementary Material


