# Supplementary material for: The Educational Gradient in the Adherence to the Healthy Nordic Food Index Among Adult Men and Women in Tromsø: The Tromsø study 2015–2016
Source: Food Nutr Res. 2026 Mar 4;70:10.29219/fnr.v70.12632. doi: 10.29219/fnr.v70.12632 (PMC13054935; doi:10.29219/fnr.v70.12632)
Supplement: Supplementary file 1 [file FNR-70-12632-s1.docx]

| **Index Food category** | | **Description of food items** | |
| --- | --- | --- | --- |
| **Fish** |  | |  |
| Fish as a main course | ·                  Cod/pollock/haddock/catfish/redfish boiled | |  |
|  | ·                  Cod/pollock/haddock/catfish/redfish fried | |  |
|  | ·                  Herring fresh/smoked/cured | |  |
|  | ·                  Mackerel fresh/smoked | |  |
|  | ·                  Fish salmon/trout, boiled/fried | |  |
|  |  | |  |
| Fish Spread | ·                  Spread caviar | |  |
|  | ·                  Spread caviar Svolvarpostei | |  |
|  | ·                  Spread mackerel in tomato sauce | |  |
|  | ·                  Spread smoked/gravlax salmon/trout | |  |
|  | ·                  Spread sardines/pickled herring/anchovy | |  |
|  | ·                  Spread tuna fish | |  |
|  |  | |  |
| **Cabbage** | ·                  Cabbage | |  |
|  | ·                  Cauliflower | |  |
|  | ·                  Brussels sprout | |  |
|  | ·                  Broccoli | |  |
|  |  | |  |
| **Whole-grain bread** | ·                  Whole-grain bread 50% | |  |
|  | ·                  Wholegrain bread 50-100% | |  |
|  | ·                  Whole-grain crispbread | |  |
|  |  | |  |
| **Apples/Pears** | ·                  Apples | |  |
|  | ·                  Pears | |  |
|  |  | |  |
| **Root vegetables** | ·                  Carrots | |  |
|  | ·                  Swede | |  |
|  |  | |  |
| **Oatmeal** | ·                  Porridge/Oatmeal | |  |
|  | ·                  Oatmeal/4-grain | |  |
|  | ·                  Cereal rice/oats puffed | |  |

Table S1. Foods included in the calculation of the 6 food items of the HNFI

Table S2a. Complete Case Full Models for Men

| **Variables** | **Model 1:** | | **Model 2:** | | **Model 3:** | | **Model 4:** | | **Model 5:** | |
| --- | --- | --- | --- | --- | --- | --- | --- | --- | --- | --- |
|  | Education + Confounder (Age) | | Model 1 + Household Income | | Model 1 + Subjective Occupational Social Status | | Model 1 + Self-rated Health | | Model 1 + All Intermediate Variables | |
| **HNFI** | **Medium** | **High** | **Medium** | **High** | **Medium** | **High** | **Medium** | **High** | **Medium** | **High** |
| **Education** | | | | | | | | | | |
| Primary/ Partly secondary | 1.0 (ref) | 1 | 1 | 1 | 1 | 1 | 1 | 1 | 1 | 1 |
| Upper-Secondary | 1.08 [0.9-1.31] | 1.34 [ 1.10-1.63] | 1.0 [0.83-1.22] | 1.2 [0.99-1.47] | 1.10 [0.91-1.33] | 1.32 [1.09-1.61] | 1.06 [0.87-1.28] | 1.28 [1.05-1.56] | 1.01 [0.83-1.22] | 1.18 [0.96-1.44] |
| Tertiary/Short | 1.33 [1.08-1.65] | 1.89 [1.52- 2.34] | 1.16 [0.93-1.44] | 1.56 [1.25-1.95] | 1.37 [1.10-1.69] | 1.84 [1.48-2.3] | 1.29 [1.04-1.59] | 1.79 [1.44-2.23] | 1.18 [0.95-1.47] | 1.53 [1.22-1.93] |
| Tertiary/Long | 1.28 [1.04-1.57] | 2.1 [1.71-2.59] | 1.07 [0.86-1.33] | 1.65 [1.32-2.07] | 1.32 [1.06-1.64] | 2.03 [1.63-2.53] | 1.2 [0.97-1.47] | 1.87 [1.51-2.31] | 1.09 [0.87-1.36] | 1.56 [1.24-1.97] |
| **Age** | 1.01 [1.01-1.02] | 1.03 [1.02-1.03] | 1.02 [1.01-1.03] | 1.03 [1.03-1.04] | 1.01 [1.01-1.02] | 1.02[1.02-1.03] | 1.01 [1.01-1.02] | 1.03 [1.02-1.03] | 1.02[1.01-1.03] | 1.03 [1.03-1.04] |
| **Household Income** | | | | | | | | | | |
| Low |  |  | 1 | 1 |  |  |  |  | 1 | 1 |
| Lower-Middle |  |  | 1.25 [1.03-1.53 | 1.52 [1.24-1.88] |  |  |  |  | 1.24 [1.01-1.51] | 1.48 [1.2-1.82] |
| Upper-Middle |  |  | 1.68 [1.34-2.1] | 2.06 [1.64-2.6] |  |  |  |  | 1.65 [1.32-2.07] | 1.96 [1.55-2.48] |
| High |  |  | 1.74 [1.38-2.2] | 2.18 [1.71-2.78] |  |  |  |  | 1.71 [1.34-2.18] | 1.98 [1.54-2.55] |
| **Subjective Occupational Social Status** | | | | | | | | | | |
| Low |  |  |  |  | 1 | 1 |  |  | 1 | 1 |
| Neither high nor low |  |  |  |  | 0.8 [0.6-1.07] | 1.13 [0.82-1.55] |  |  | 0.73 [0.54-0.98] | 0.99 [0.72-1.38] |
| High |  |  |  |  | 0.7 [0.58-1.06] | 1.19 [0.86-1.65] |  |  | 0.67 [0.5-0.92] | 0.97 [0.69-1.35] |
| **Self-rated Health** | | | | | | | | | | |
| Bad |  |  |  |  |  |  | 1 | 1 | 1 | 1 |
| Neither good nor bad |  |  |  |  |  |  | 1.3 [0.944-1.78] | 1.5 [1.06-2.11] | 1.2 [0.87-1.66] | 1.35 [0.96-1.91] |
| Good |  |  |  |  |  |  | 1.54 [1.13-2.09] | 1.85 [1.33-2.56] | 1.4 [1.02-1.91] | 1.6 [1.15-2.24] |
| Excellent |  |  |  |  |  |  | 1.73 [1.21-2.49] | 2.7 [1.85-3.95] | 1.55 [1.07-2.25] | 2.29 [1.56-3.36] |

Table S2b. Complete Case Full Models for Women

| **Variables** | **Model 1:** | | **Model 2:** | | **Model 3:** | | **Model 4:** | | **Model 5:** | |
| --- | --- | --- | --- | --- | --- | --- | --- | --- | --- | --- |
|  | Education + Confounder (Age) | | Model 1 + Household Income | | Model 1 + Subjective Occupational Social Status | | Model 1 + Self-rated Health | | Model 1 + All Intermediate Variables | |
| **HNFI** | **Medium** | **High** | **Medium** | **High** | **Medium** | **High** | **Medium** | **High** | **Medium** | **High** |
| **Education** | | | | | | | | | | |
| Primary/ Partly secondary | 1.0 (ref) | 1 | 1 | 1 | 1 | 1 | 1 | 1 | 1 | 1 |
| Upper-Secondary | 1.02 [0.84-1.24] | 1.24 [1.02-1.51] | 1.01 [0.83-1.23] | 1.2 [0.99-1.47] | 1.03 [0.85-1.25] | 1.26 [1.03-1.53] | 1.01 [0.83-1.22] | 1.21 [1-1.48] | 1.01 [0.83-1.22] | 1.2 [0.99-1.47] |
| Tertiary/Short | 1.47 [1.18-1.84] | 1.85 [1.48-2.33] | 1.42 [1.13-1.79] | 1.73 [1.37-2.18] | 1.48 [1.18-1.86] | 1.87 [1.48-2.36] | 1.42 [1.14-1.78] | 1.76[1.4-2.21] | 1.41 [1.12-1.78] | 1.72[1.36-2.18] |
| Tertiary/Long | 1.63 [1.33-1.98] | 2.13 [1.74-2.6] | 1.54 [1.24-1.9] | 1.93 [1.56-2.4] | 1.62 [1.32-2.01] | 2.13 [1.72-2.63] | 1.54 [1.26-1.89] | 1.96 [1.6-2.41] | 1.52 [1.22-1.89] | 1.89 [1.51-2.37] |
| **Age** | 1.02 [1.01-1.02] | 1.04 [1.03-1.04] | 1.02 [1.01-1.02] | 1.04 [1.03-1.05] | 1.02 [1.01-1.02] | 1.04 [1.03-1.04] | 1.02 [1.01-1.02] | 1.04 [1.03-1.04] | 1.02 [1.01-1.02] | 1.04 [1.03-1.05] |
| **Household Income** | | | | | | | | | | |
| Low |  |  | 1 | 1 |  |  |  |  | 1 | 1 |
| Lower-Middle |  |  | 1.14 [0.95-1.37] | 1.27 [1.06-1.53] |  |  |  |  | 1.11 [0.92-1.33] | 1.23 [1.02-1.49] |
| Upper-Middle |  |  | 1.11 [0.9-1.36] | 1.29 [1.04-1.59] |  |  |  |  | 1.06 [0.86-1.31] | 1.23 [0.99-1.52] |
| High |  |  | 1.19 [0.95-1.49] | 1.34 [1.06-1.68] |  |  |  |  | 1.12 [0.89-1.41] | 1.22 [0.96-1.54] |
| **Subjective Occupational Social Status** | | | | | | | | | | |
| Low |  |  |  |  | 1 | 1 |  |  | 1 | 1 |
| Neither high nor low |  |  |  |  | 0.9 [0.69-1.17] | 0.77 [0.59-1.01] |  |  | 0.86 [0.66-1.12] | 0.72 [0.55-0.95] |
| High |  |  |  |  | 0.93 [0.7-1.24] | 0.84 [0.63-1.12] |  |  | 0.87 [0.65-1.16] | 0.76 [0.57-1.01] |
| **Self-rated Health** | | | | | | | | | | |
| Bad |  |  |  |  |  |  | 1 | 1 | 1 | 1 |
| Neither good nor bad |  |  |  |  |  |  | 1.47 [1.1-1.95] | 1.63 [1.21-2.19] | 1.46 [1.09-1.94] | 1.6 [1.19-2.16] |
| Good |  |  |  |  |  |  | 1.62 [1.23-2.11] | 1.85 [1.39-2.45] | 1.6 [1.22-2.1] | 1.81 [1.36-2.41] |
| Excellent |  |  |  |  |  |  | 1.76 [1.29-2.39] | 2.29 [1.67-3.15] | 1.73 [1.27-2.37] | 2.24 [1.62-3.09] |

Table S3. Bivariate Analysis between the HNFI and the independent variables

|  | **Men** | | | | **p-value** | **Women** | | | | **p-value** |
| --- | --- | --- | --- | --- | --- | --- | --- | --- | --- | --- |
|  | **Healthy Nordic Food Index Adherence Categories** | | | |  | **Healthy Nordic Food Index Adherence Categories** | | | |  |
|  | **Total (n=6986)** | **Low (n=572)** | **Medium (n=2818)** | **High (n=3596)** |  | **Total (n=8160)** | **Low (n=1316)** | **Medium (n=3481)** | **High (n=3363)** |  |
| **Education** |  |  |  |  | 0.002 |  |  |  |  | <0.001 |
| Primary/Partly secondary | 1536 (22.0%) | 145 (25.3%) | 664 (23.6%) | 727 (20.2%) |  | 1998 (24.5%) | 362 (27.5%) | 855 (24.6%) | 781 (23.2%) |  |
| Upper Secondary | 2035 (29.1%) | 168 (29.4%) | 835 (29.6%) | 1032 (28.7%) |  | 2026 (24.8%) | 382 (29.0%) | 809 (23.2%) | 835 (24.8%) |  |
| Tertiary/Short | 1499 (21.5%) | 116 (20.3%) | 582 (20.7%) | 801 (22.3%) |  | 1382 (16.9%) | 196 (14.9%) | 605 (17.4%) | 581 (17.3%) |  |
| Tertiary/Long | 1804 (25.8%) | 129 (22.6%) | 693 (24.6%) | 982 (27.3%) |  | 2611  (32%) | 345 (26.2%) | 1143 (32.8%) | 1123 (33.4%) |  |
| Missing | 112 (1.6%) | 14 (2.4%) | 44 (1.6%) | 54 (1.5%) |  | 143 (1.8%) | 31 (2.4%) | 69 (2.0%) | 43 (1.3%) |  |
| **Age Mean (SD)** | 59.1 (11.3) | 57.3 (12.6) | 58.2 (11.4) | 60.0 (10.9) | <0.001 |  | 56.9 (11.9) | 58.0 (11.5) | 59.4 (10.7) | <0.001 |
| **Household Income** |  |  |  |  | <0.001 |  |  |  |  | 0.078 |
| Low | 1279 (18.3%) | 149 (26.0%) | 551 (19.6%) | 579 (16.1%) |  | 2116 (25.9%) | 375 (28.5%) | 887 (25.5%) | 854 (25.4%) |  |
| Lower-Middle | 2035 (29.1%) | 159 (27.8%) | 809 (28.7%) | 1067 (29.7%) |  | 2350 (28.8%) | 358 (27.2%) | 982 (28.2%) | 1010 (30.0%) |  |
| Upper-Middle | 1678 (24.0%) | 119 (20.8%) | 673 (23.9%) | 886 (24.6%) |  | 1612 (19.8%) | 259 (19.7%) | 685 (19.7%) | 668 (19.9%) |  |
| High | 1839 (26.3%) | 124 (21.7%) | 719 (25.5%) | 996 (27.7%) |  | 1621 (19.9%) | 237 (18.0%) | 726 (20.9%) | 658 (19.6%) |  |
| Missing | 155 (2.2%) | 21 (3.7%) | 66 (2.3%) | 68 (1.9%) |  | 461 (5.6%) | 87 (6.6%) | 201 (5.8%) | 173(5.1%) |  |
| **Subjective Occupational Social Status** |  |  |  |  | <0.001 |  |  |  |  | 0.019 |
| Low | 385 (5.5%) | 30 (5.2%) | 189 (6.7%) | 166 (4.6%) |  | 590 (7.2%) | 98 (7.4%) | 245 (7.0%) | 247 (7.3%) |  |
| Neither high nor low | 3174 (45.4%) | 275 (48.1%) | 1332 (47.3%) | 1567 (43.6%) |  | 4348 (53.3%) | 738 (56.1%) | 1859 (53.4%) | 1751 (52.1%) |  |
| High | 3282 (47.0%) | 247 (43.2%) | 1243 (44.1%) | 1792 (49.8%) |  | 2987 (36.6%) | 425 (32.3%) | 1280 (36.8%) | 1282 (38.1%) |  |
| Missing | 145 (2.1%) | 20 (3.5%) | 54 (1.9%) | 71 (2.0%) |  | 235 (2.9%) | 55 (4.2%) | 97 (2.8%) | 83 (2.5%) |  |
| **Self-rated Health** |  |  |  |  | <0.001 |  |  |  |  | <0.001 |
| Bad | 309 (4.4%) | 40 (7.0%) | 147 (5.2%) | 122 (3.4%) |  | 450 (5.5%) | 114 (8.7%) | 189 (5.4%) | 147 (4.4%) |  |
| Neither good nor bad | 1784 (25.5%) | 161 (28.1%) | 753 (26.7%) | 870 (24.2%) |  | 2083 (25.5%) | 361 (27.4%) | 900 (25.9%) | 822 (24.4%) |  |
| Good | 3947 (56.5%) | 307 (53.7%) | 1581 (56.1%) | 2059 (57.3%) |  | 4198 (51.4%) | 645 (49.0%) | 1796 (51.6%) | 1757 (52.2%) |  |
| Excellent | 907 (13.0%) | 57 (10.0%) | 318 (11.3%) | 532 (14.8%) |  | 1347 (16.5%) | 181 (13.8%) | 560 (16.1%) | 606 (18.0%) |  |
| Missing | 39 (0.6%) | 7 (1.2%) | 19 (0.7%) | 13 (0.4%) |  | 82 (1.0%) | 15 (1.1%) | 36 (1.0%) | 31 (0.9%) |  |

Table S4a. Full Models with Imputed Data for Men

| **Variables** | **Model 1:** | | **Model 2:** | | **Model 3:** | | **Model 4:** | | **Model 5:** | |
| --- | --- | --- | --- | --- | --- | --- | --- | --- | --- | --- |
|  | Education + Confounder (Age) | | Model 1 + Household Income | | Model 1 + Subjective Occupational Social Status | | Model 1 + Self-rated health | | Model 1 + All Intermediate Variables | |
| **HNFI** | **Medium** | **High** | **Medium** | **High** | **Medium** | **High** | **Medium** | **High** | **Medium** | **High** |
| **Education** | | | | | | | | | | |
| Primary/Partly secondary | 1.0 (ref) | 1 | 1 | 1 | 1 | 1 | 1 | 1 | 1 | 1 |
| Upper-Secondary | 1.17 [0.91-1.49] | 1.45 [1.13-1.86] | 1.06 [0.83-1.36] | 1.24 [0.97-1.6] | 1.19 [0.93-1.52] | 1.45 [1.13-1.85] | 1.14 [0.89-1.46] | 1.38 [1.08-1.77] | 1.07 [0.83-1.38] | 1.22 [0.95-1.57] |
| Tertiary/Short | 1.19 [0.91-1.56] | 1.66 [1.27-2.17] | 1 [0.75-1.32] | 1.24 [0.94-1.65] | 1.23 [0.93-1.63] | 1.62 [1.23-2.13] | 1.16 [0.88-1.53] | 1.56 [1.19-2.05] | 1.03 [0.77-1.38] | 1.23 [0.92-1.63] |
| Tertiary/Long | 1.29 [0.99-1.68] | 1.92 [1.47-2.5] | 1.04 [0.78-1.38] | 1.34 [1.01-1.78] | 1.35 [1.01-1.79] | 1.83 [1.38-2.42] | 1.23 [0.94-1.62] | 1.7 [1.3-2.22] | 1.08 [0.8-1.46] | 1.27 [0.94-1.71] |
| **Age** | 1.01[1.0-1.02] | 1.03[1.02-1.04] | 1.02[1.01-1.03] | 1.04[1.03-1.05] | 1.01 [1.0-1.01] | 1.03[1.01-1.03] | 1.01[1.0-1.01] | 1.02[1.02-1.03] | 1.02[1.0-1.02] | 1.04[1.03-1.05] |
|  | | | | | | | | | | |
| Low |  |  | 1 | 1 |  |  |  |  | 1 | 1 |
| Lower-Middle |  |  | 1.53 [1.19-1.98] | 2.08 [1.61-2.69] |  |  |  |  | 1.53[1.18-1.98] | 2.02[1.56-2.62] |
| Upper-Middle |  |  | 1.89 [1.41-2.52] | 2.85 [2.14-3.8] |  |  |  |  | 1.9[1.41-2.54] | 2.72[2.03-3.65] |
| High |  |  | 1.99 [1.46-2.69] | 3.16 [2.33-4.28] |  |  |  |  | 2.03[1.48-2.79] | 2.9[2.12-3.98] |
| **Subjective Occupational Social Status** | | | | | | | | | | |
| Low |  |  |  |  | 1 | 1 |  |  | 1 | 1 |
| Neither high nor low |  |  |  |  | 0.71[0.47-1.07] | 0.87[0.57-1.31] |  |  | 0.64[0.42-0.96] | 0.73[0.48-1.11] |
| High |  |  |  |  | 0.7 [0.46-1.06] | 0.99[0.65-1.5] |  |  | 0.59[0.38-0.9] | 0.65[0.49-1.16] |
| **Self-rated Health** | | | | | | | | | | |
| Bad |  |  |  |  |  |  | 1 | 1 | 1 | 1 |
| Neither good nor bad |  |  |  |  |  |  | 1.26[0.85-1.86] | 1.75[1.17-2.61] | 1.13[0.76-1.68] | 1.5[1.0-2.25] |
| Good |  |  |  |  |  |  | 1.39[0.96-2.02 | 2.19[1.5-3.21] | 1.22[0.84-1.79] | 1.78[1.2-2.62] |
| Excellent |  |  |  |  |  |  | 1.48[0.94-2.34] | 3.02[1.91-4.78] | 1.28[0.81-2.05] | 2.35[1.47-3.76] |

Table S4b. Full Models with Imputed Data for Women

| **Variables** | **Model 1:** | | **Model 2:** | | **Model 3:** | | **Model 4:** | | **Model 5:** | |
| --- | --- | --- | --- | --- | --- | --- | --- | --- | --- | --- |
|  | Education + Confounder (Age) | | Model 1 + Household Income | | Model 1 + Subjective Occupational Social Status | | Model 1 + Self-rated health | | Model 1 + All Intermediate Variables | |
| **HNFI** | **Medium** | **High** | **Medium** | **High** | **Medium** | **High** | **Medium** | **High** | **Medium** | **High** |
| **Education** | | | | | | | | | | |
| Primary/Partly secondary | 1.0 (ref) | 1 | 1 | 1 | 1 | 1 | 1 | 1 | 1 | 1 |
| Upper-Secondary | 1.04 [0.87-1.25] | 1.34 [1.12-1.61] | 1.02 [0.85-1.22] | 1.29 [1.07-1.55] | 1.04 [0.87-1.25] | 1.35 [1.13-1.63] | 1.02 [0.85-1.23] | 1.3 [1.09-1.57] | 1.01 [0.84-1.22] | 1.28 [1.06-1.54] |
| Tertiary/Short | 1.58 [1.28-1.96] | 1.99 [1.6-2.47] | 1.5 [1.21-1.87] | 1.84 [1.47-2.3] | 1.59 [1.28-1.98] | 2 [1.61-2.5] | 1.53 [1.23-1.9] | 1.87 [1.5-2.33] | 1.5 [1.2-1.87] | 1.81 [1.44-2.27] |
| Tertiary/Long | 1.76 [1.46-2.13] | 2.35 [1.94-2.85] | 1.64 [1.33-2] | 2.11 [1.72-2.59] | 1.76 [1.44-2.15] | 2.34 [1.91-2.87] | 1.68 [1.39-2.04] | 2.15 [1.77-2.62] | 1.62 [1.31-2] | 2.05 [1.65-2.54] |
| **Age** | 1.01[1.0-1.02] | 1.03[1.02-1.04] | 1.01[1.01-1.02] | 1.03[1.02-1.04] | 1.01 [1.01-1.02] | 1.03[1.02-1.04] | 1.01[1.01-1.02] | 1.03[1.02-1.04] | 1.02[1.01-1.02] | 1.03[1.03-1.04] |
| **Household Income** | | | | | | | | | | |
| Low |  |  | 1 | 1 |  |  |  |  | 1 | 1 |
| Lower-Middle |  |  | 1.17 [0.98-1.4] | 1.33 [1.11-1.6] |  |  |  |  | 1.15[0.96-1.37] | 1.29[1.08-1.54] |
| Upper-Middle |  |  | 1.19 [0.97-1.46] | 1.37 [1.11-1.69] |  |  |  |  | 1.15[0.94-1.42] | 1.3[1.06-1.61] |
| High |  |  | 1.25 [1.0-1.56] | 1.36 [1.09-1.7] |  |  |  |  | 1.19[0.95-1.49] | 1.23[0.98-1.55] |
| **Perceived Occupation Social Status** | | | | | | | | | | |
| Low |  |  |  |  | 1 | 1 |  |  | 1 | 1 |
| Neither high nor low |  |  |  |  | 0.89[0.69-1.14] | 0.78[0.61-1.01] |  |  | 0.85[0.66-1.10] | 0.74[0.57-0.96] |
| High |  |  |  |  | 0.93[0.7-1.22] | 0.86[0.65-1.13] |  |  | 0.87[0.66-1.15] | 0.78[0.59-1.03] |
| **Perception of Health** | | | | | | | | | | |
| Bad |  |  |  |  |  |  | 1 | 1 | 1 | 1 |
| Neither good nor bad |  |  |  |  |  |  | 1.45[1.16-1.89] | 1.65[1.25-2.17] | 1.44[1.10-1.87] | 1.62[1.23-2.14] |
| Good |  |  |  |  |  |  | 1.57[1.22-2.02] | 1.94[1.49-2.52] | 1.54[1.2-1.99] | 1.89[1.45-2.47] |
| Excellent |  |  |  |  |  |  | 1.69[1.26-2.26] | 2.37[1.75-3.2] | 1.65[1.22-2.21] | 2.31[1.7-3.13] |

Table S5. Models adjusted for household size for men and women

| **Models adjusted for household size** | | | | |
| --- | --- | --- | --- | --- |
|  | **Model 2:**  **Education + Confounder (Age) + Household Income + Household Size** | | **Model 5:**  **Education + Confounder (Age) + Household Income + Subjective Occupational Social Status + Self-rated health + Household Size** | |
| **HNFI** | Medium | High | Medium | High |
| **Education -Men** | | | | |
| Primary/Partly secondary | 1 | 1 | 1 | 1 |
| Upper Secondary | 1.06 [0.82-1.36] | 1.24 [0.96-1.59] | 1.07 [0.83-1.38] | 1.22 [0.94-1.57] |
| Tertiary Short | 1 [0.74-1.31] | 1.24 [0.93-1.64] | 1.02 [0.77-1.36] | 1.22 [0.92-1.63] |
| Tertiary Long | 1.03 [0.77-1.38] | 1.34 [1.01-1.78] | 1.08 [0.8-1.46] | 1.27 [0.94-1.71] |
| **Education- Women** | | | | |
| Primary/Partly secondary | 1 | 1 | 1 | 1 |
| Upper Secondary | 1.07 [0.83-1.38] | 1.22 [0.94-1.57] | 1 [0.84-1.21] | 1.26 [1.05-1.52] |
| Tertiary Short | 1.02 [0.77-1.36] | 1.22 [0.92-1.63] | 1.49 [1.19-1.86] | 1.79 [1.42-2.25] |
| Tertiary Long | 1.08 [0.8-1.46] | 1.27 [0.94-1.71] | 1.61 [1.3-1.99] | 2.03 [1.63-2.51] |
